# Mindfulness Improves Emotion Regulation and Executive Control on Bereaved Individuals: An fMRI Study

**DOI:** 10.3389/fnhum.2018.00541

**Published:** 2019-01-28

**Authors:** Feng-Ying Huang, Ai-Ling Hsu, Li-Ming Hsu, Jaw-Shiun Tsai, Chih-Mao Huang, Yi-Ping Chao, Tzung-Jeng Hwang, Changwei W. Wu

**Affiliations:** ^1^Department of Education, College of Education, National Taipei University of Education, Taipei, Taiwan; ^2^Department of Radiology, Wan Fang Hospital, Taipei Medical University, Taipei, Taiwan; ^3^Department of Radiology and Biomedical Research Imaging Center, School of Medicine, University of North Carolina at Chapel Hill, Chapel Hill, NC, United States; ^4^Department of Family Medicine, College of Medicine and Hospital, National Taiwan University, Taipei, Taiwan; ^5^Center for Complementary and Integrated Medicine, National Taiwan University Hospital, Taipei, Taiwan; ^6^Department of Biological Science and Technology, College of Biological Science and Technology, National Chiao Tung University, Hsinchu, Taiwan; ^7^Graduate Institute of Medical Mechatronics, Chang Gung University, Taoyuan, Taiwan; ^8^Department of Psychiatry, National Taiwan University Hospital and College of Medicine, Taipei, Taiwan; ^9^Graduate Institute of Mind, Brain and Consciousness, Taipei Medical University, Taipei, Taiwan; ^10^Research Center of Brain and Consciousness, Shuang Ho Hospital, New Taipei, Taiwan

**Keywords:** bereavement grief, mindfulness-based cognitive therapy (MBCT), emotion regulation, executive control, functional magnetic resonance imaging (fMRI)

## Abstract

The grief of bereavement is recognized as a severe psychosocial stressor that can trigger a variety of mental and physical disorders, and the long-lasting unresolved grief has a detrimental effect on brain functionality. Literature has documented mindfulness-based cognitive therapy (MBCT) as an efficient treatment for improving well-being, specifically related to the mood and cognition, in a variety of populations. However, little attention has been devoted to neural mechanisms with regard to bereaved individuals’ cognition after MBCT intervention. In this study, we recruited 23 bereaved participants who lost a significant relative within 6 months to 4 years to attend 8-week MBCT course. We used self-reporting questionnaires to measure emotion regulation and functional magnetic resonance imaging (fMRI) with the numerical Stroop task to evaluate the MBCT effect on executive control among the bereaved participants. The self-reported questionnaires showed improvements on mindfulness and reductions in grief, difficulties in emotion regulation, anxiety, and depression after the MBCT intervention. The fMRI analysis demonstrated two scenarios: (1) the activity of the fronto-parietal network slightly declined accompanied with significant improvements in the reaction time of incongruent trials; (2) the activities in the posterior cingulate cortex and thalamus were positively associated with the Texas Revised Inventory of Grief, implying emotional interferences on cognitive functions. Results indicated that MBCT facilitated the executive control function by alleviating the emotional interferences over the cognitive functions and suggested that the 8-week MBCT intervention significantly improved both executive control and emotion regulation in bereaved individuals.

## Introduction

Suffering the death of a loved one is recognized as a severe psychological stressor that results in a time of excessive risk of mental and physical problems (Stroebe et al., 2007; Buckley et al., 2010). The emotional response to bereavement, mainly referred to as grief, incorporates diverse biological, psychological, and behavioral symptoms. Bereavement grief has been associated with symptoms of depression, distress, and anxiety (Byrne and Raphael, 1997; Zisook et al., 1997; Taylor et al., 1999). These negative impacts thus complicated bereaved individuals’ heath problems (Beem et al., 2000; Buckley et al., 2010; Assareh et al., 2015) and potentially cause a reduction in cognitive functions (Clayton et al., 1971; Gündel et al., 2003; Rosnick et al., 2010). Furthermore, considerable evidence has also shown increased mortality and morbidity rates in the early months of bereavement (Young et al., 1963; Lichtenstein et al., 1998; Christakis and Iwashyna, 2003; Hart et al., 2007), although widowers have been identified as possibly most at risk (Kaprio et al., 1987; Buckley et al., 2010).

Mindfulness is commonly defined as paying attention to the internal and external experiences occurring in the present moment, without judgment or reaction (Kabat-Zinn, 1994). Fostering the ability to be focused on the present moment and detached observers of our inner cognition or emotions that are adopted during mindfulness training is thought to promote cognitive function and to enhance objective and adaptive strategies of responding to emotional or cognitive triggers (Bishop, 2004; Kang et al., 2013). Neuroimaging research of mindfulness also demonstrated that mindfulness can reduce the activity of the amygdala and increases the thickness of the cerebral cortex (Hölzel et al., 2011; Kral et al., 2018). Several key elements such as attention regulation, body awareness, emotion regulation, acceptance, self-transcendence, and cognitive flexibility are considered to be developed effectively by mindfulness training (Shapiro et al., 2006; Moore and Malinowski, 2009; Hölzel et al., 2011; Vago and Silbersweig, 2012). Research on mindfulness has been well documented to be beneficial for people’s emotional regulation (Hölzel et al., 2011; Teper and Inzlicht, 2013; Roemer et al., 2015), such as in psychotherapy for Major Depressive Disorder (Ma and Teasdale, 2004), Anxiety Disorder (Goldin and Gross, 2010). The mindfulness-based intervention has been found to decrease relapse or recurrence of depression (Teasdale et al., 2000), with equivalent effectiveness to antidepressant treatment (Kuyken et al., 2015), to strengthen the ability of emotion regulation, such as the management of anxiety symptoms (Hoge et al., 2013) and stress (Shapiro et al., 2005). Remarkably, mindfulness interventions have been reported to improve executive functions (Teper and Inzlicht, 2013), such as working memory and sustained attention and attention switching (Chambers et al., 2007; Jha et al., 2007; Zeidan et al., 2010). In addition, based on a review paper conducted by Gallant (2016), the benefit of mindfulness on inhibition is more consistently identified than other executive functions.

Given the reviewed literature on beneficial effects of mindfulness, mindfulness-based cognitive therapy (MBCT) is developed accordingly as a group-based intervention that teaches participants (1) keeping awareness without fusion with contents of cognition or psychophysical experiences; (2) observing the emotional arousals corresponding to the stressful events, such as grief and negative thought specifically evoked by the death of relative one; (3) accepting the emotion without judgments and then switching attention to a neutral object (e.g., the body sensations or breath) (Teasdale et al., 2000; Segal et al., 2002, 2013). MBCT has been proven a great success in assisting patients with depressive and bipolar disorders for exercising mood regulation, and broad attention and inhibitory control (Kuyken et al., 2015; Lovas and Schuman-Olivier, 2018). Although MBCT provides a positive impact on bereaved individuals, the remaining issues are whether emotional disentanglements improve cognitive functions and what the underlying neurophysiological basis is. Henceforth, we examined MBCT modulations on bereaved individuals in terms of their neural activities using a cognitive-fMRI experiment with the numerical Stroop task, and emotion regulation abilities using a series of self-reported questionnaires. We therefore hypothesized that the cognitive function in bereaved participants would be improved following the MBCT training, where the same participants were evaluated before and after the MBCT intervention.

## Materials and Methods

We used the experimental design with both self-reported questionnaires and fMRI sessions before the 8-week MBCT training (***Pre***) and after MBCT intervention (***Post***), respectively. A within-subject design was employed to compare the cognitive performance and fMRI activation changes related to 8-week MBCT intervention.

### Participants

Twenty-three bereaved subjects (21 females and 2 males) aged between 25 and 66 (mean = 48.35, *SD* = 11.14) having lost at least one significant relative within 6 months to 4 years and having self-reported unresolved grief participated in the study. The participants were recruited by flyers distributed throughout the Taipei city and by advertising in National Taipei University in Education’s Internet forums. All Participants were native Mandarin speakers. Participants would receive a free 8-week MBCT course. Exclusion criteria were: previous mindfulness meditation experience, history of psychological/psychiatric disease, use of prescription drugs, and MR incompatibility. Four of these were excluded for the following reasons: two moved out of city before the MBCT intervention; one dropped out in the middle of intervention; another one could not make it to the MRI scan after intervention. Complete data sets were thus available from 19 and 20 participants for fMRI scanning and questionnaire analysis, respectively. Written informed consent was obtained from each participant in accordance with the guidelines and the study protocol approved by the Research Ethics Office of National Taiwan University.

### Self-Reported Questionnaires

The severity of grief was assessed by Texas Revised Inventory of Grief (TRIG) (Faschingbauer et al., 1987), which is composed of the parts of past behaviors and present feelings. Since the aim of this study is to evaluate the effects of MBCT intervention on the present cognitive and emotion reactions, only present grief part of the inventory was used in the present study. In testing the tendency of anxiety level and the severity of depressive symptoms, the Generalized Anxiety Disorder-7 (GAD-7) (Spitzer et al., 2006) and the 18-item Taiwan Depression Scale (Lee et al., 2000) were adopted, respectively. To further evaluate the degree of difficulty of emotion regulation, Difficulties in Emotion Regulation Scale (DERS) (Gratz and Roemer, 2004) was employed.

The Five Facet Mindfulness Questionnaire (FFMQ) is a widely used 39-item questionnaire sensitively assessing the traits that are cultivated by mindfulness (Baer et al., 2008). Since the validity and reliability of the Taiwanese version of FFMQ has been verified elsewhere (Huang et al., 2015), we directly employed the T-FFMQ and reported the total scores in this study.

### Mindfulness-Based Cognitive Therapy (MBCT)

The intervention followed the group-based MBCT program (Segal et al., 2013), which consists of once-weekly meetings (with a duration of 2.5 h) plus daily home practice (30–40 min a day) in the 8 weeks of the course. During the group sessions, participants were led by a group therapist with skills training and in-class practice in (1) guided meditation, (2) experiential exercises, and (3) discussions of the participants’ daily practices. The group therapist, the first author of this study, is a certificated grief therapist and has more than 3,200 h of experience in facilitating mindfulness group interventions. Specific in-class guided meditation included body scans, sitting meditation, compassion meditation, and yoga. In addition to the group sessions, participants were instructed to practice mindfulness exercises aided by standard audio-recordings throughout the day, and to record their daily practice times at home, which were evaluated in the weekly course session. Furthermore, an extra 2-h introduction session of “acknowledgment of grief and theory of psycho-physical reactions to loss” specifically designed for bereaved individuals was added before the standard MBCT program.

### Experimental Tasks

The fMRI session was divided into three sessions with two sessions of numerical Stroop tasks. Stimuli were presented via E-prime (Psychology Software Tools, Pittsburgh, PA, United States) with a back-projection system. The visual stimuli were presented in 800 × 600 resolution. Participants viewed the stimuli using a mirror mounted on the head-coil and the viewing field was 8.4° (H) by 6.3° (V) at a viewing distance of 420 cm. Participants were instructed to respond with a button press using the index and middle fingers of their right hand (Lumina response pad; Cedrus, San Pedro, CA, United States).

We conducted the numerical Stroop tasks in the current study to assess the executive control function for bereaved participants. Two types of magnitude judgments were included in this task: a physical size task and a numerical magnitude task. In the physical size task, participants were presented with a pair of digits and were instructed to judge which digit was physically larger while ignoring the numerical magnitude of the digits. On the other hand, in the numerical magnitude task, participants were viewing a pair of digits and were asked to indicate which was numerically larger while ignoring their physical size. In both tasks, the individual digits between 1 and 9 excluding 5 were used to create the digit pairs, and digit pairs were presented in Arial font with two different font sizes (55 and 73) to manipulate the physical size of the items. For each session in numerical Stroop task, four blocks were designed with two blocks of congruent condition and two blocks of incongruent condition, and the presentation sequence was randomized to minimize the fatigue effects. Each block started with a 30-s fixation-cross resting period and 36-s presentation of digit-pair trials, with each trial consisting of a 1-s fixation and 1-s presentation of stimulus. The participants were asked to make judgments by pressing buttons within the 1-s stimuli presentation periods. In congruent blocks, the digit that was larger in magnitude was also larger in physical size. In incongruent trials, the digit that was larger in magnitude was smaller in the physical size. The total acquisition time for the numerical Stroop task was 264 s.

### MRI Data Acquisition

The MRI experiments were conducted at a 3T PRISMA scanner (Siemens, Erlangen, Germany) at the National Taiwan University. All visual stimuli were given by a projector with E-Prime software, reflected by mirror settings. To alleviate the motion artifact due to the speaking, the head position of participants was immobilized using the thermoset plastics. The scanning protocol included one high-resolution T_1_-weighted anatomical image using 3D-MPRAGE sequence and two functional sessions of numerical Stroop tasks using a single-shot, gradient-echo-based echo-planar imaging (GE-EPI) sequence. The detailed parameters for 3D-MPRAGE sequence was listed below: 192 × 192 × 176 matrix size; 1 mm × 1 mm × 1 mm in-plane resolution; 900 ms inversion time; repetition time (TR) = 1,900 ms, echo time (TE) = 2.28 ms; flip angle (FA) = 9°; bandwidth = 200 Hz/pixel; NEX = 1. Total scan time is 5 min 21 s. Subsequently, the functional sessions shared the same geometry settings: 37 axial slices (FOV = 220 × 220 mm^2^, 64 × 64 in-plane matrix size, and 3.4 mm thickness) acquired in an interleaved manner, aligned along the anterior commissure–posterior commissure (AC-PC) line with whole-brain coverage. The GE-EPI scan protocol was using imaging parameters as follows: TR = 2 s, TE = 35 ms, FA = 84°, bandwidth = 2368 Hz/pixel and total acquisition time for each session was 264 s.

### Data Analysis

All MRI data processing were analyzed by using Analysis of Functional Neuroimaging (AFNI) software package (Cox, 1996) and FMRIB Software Library (FSL) (Jenkinson et al., 2012). The task-fMRI data sets were preprocessed including slice-timing correction, motion correction, spatial normalization into the Montreal Neurological Institute (MNI) template space, 6-mm FWHM spatial smoothing. After preprocessing, task-fMRI responses for the congruent and incongruent conditions were separately modeled by convolving a canonical hemodynamic response function (HRF) with the task paradigm and retrieved the beta value as the effect size. The design matrix in a first-level fixed-effects analysis comprises two regressors of main interest: one for intervention (pre- vs. post-MBCT) and another for condition (congruent or incongruent) contrasts. Furthermore, six additional regressors modeled the head motion in preprocessing were set as the covariates of no interest. Parameter estimates from the resulting contrast maps were then entered into a second-level random-effects analysis to identify brain regions that were significantly activated by a contrast across participants. Voxel-wise one-sample *t*-tests were conducted to detect the activated voxels associated with task conditions under both before and after the MBCT intervention. As the correction for multiple comparisons, the significance level of corrected *p* < 0.01 was set with a combination of uncorrected threshold of *p* < 0.001 and individual cluster size of 90 contiguous voxels. To further interpret the 8-week MBCT effect, we performed the voxel-wise paired sample *t*-test for each task condition. Considering the reduction of statistical sensitivity on intervention effect due to the noise amplification of substation method, the multiple comparison was conducted with an explicit brain mask, and the overall significance level of *p* < 0.05 was controlled in combination of uncorrected threshold of *p* < 0.005 and individual cluster size of 103 contiguous voxels. The group-level activation areas were served as prior knowledge for the following region-of-interest (ROI) analysis. To avoid the double-dipping problem, we extracted the ROI values based on the automated anatomical labeling (AAL) template (Tzourio-Mazoyer et al., 2002) and performed the brain-behavior correlation analysis with questionnaire scores. Furthermore, since the thalamus is generally involved in the emotion regulation (Greicius et al., 2007; Peng et al., 2012), the thalamic ROI was hypothetically selected for the correlation analysis in this study.

The primary behavioral outcome was computed as the percent increase in RT to incongruent stimuli over and above the average RT to congruent stimuli ([(incongruent-congruent)/congruent] × 100) (Colcombe et al., 2005). The percent increase measure was derived to reflect interference unbiased by differences in base RT. Only correct responses were included in the outcome measure. Paired *t*-test was conducted between the MBCT interventions in general. However, if the data distribution violated the normality assumption by Shapiro–Wilk test, the non-parametric Mann–Whitney test was used instead. In addition, a two-way repeated-measure analysis of variance (ANOVA) was performed on within-subject factors of time (pre-MBCT, post-MBCT) and conditions (congruent, incongruent).

## Results

### Results of Self-Reported Questionnaires

To examine self-reported questionnaires with regards to the effectiveness of the MBCT intervention, participants’ post-MBCT scores were compared with their pre-MBCT scores on FFMQ, TRIG, DERS, Depression and Anxiety scores by using repeat measure of *t*-test. All scores of mindfulness and psychological variables were significant at *p* < 0.01. For instance, effect of TRIG was significant at *t*(19) = -3.98, *p* < 0.001, *d* = -0.89. Similarly, T-FFMQ was significant at *t*(19) = 3.57, *p* < 0.01, *d* = 0.80. The MBCT intervention was associated with effect sizes (Cohen’s *d*) of -0.89, -0.65, -1.17, and -0.76 for alleviating grief, anxiety, depression and difficulty of emotion regulation, respectively, whereas the effect size was 0.80 for improving the mindfulness level. These robust effect sizes indicated that MBCT greatly reduced negative emotions, and increased the mindfulness level among bereaved participants. Summary of the results for each psychological variable were shown in Table 1.

**Table 1:** Descriptive and statistical overview of self-reported questionnaires between pre- and post-MBCT on grief bereavement (means and standard deviations).

Source	Pre-MBCT		Post-MBCT		*t*-test		
	*Mean*	*SD*	**Mean**	*SD*	*t*-value	*p*-value	Cohen’s *d*
TRIG	49.80	13.47	37.95	12.58	-3.98	0.001**	-0.89
GAD-7	10.30	5.32	6.50	5.31	-2.89	0.009**	-0.65
Depression	23.35	11.41	12.90	11.74	-5.23	0.000**	-1.17
DERS	103.10	19.16	88.95	19.07	-3.39	0.003**	-0.76
T-FFMQ	111.10	17.18	127.45	23.94	3.57	0.002**	0.80

*^∗∗^p < 0.01.*

This finding indicated that the mindfulness intervention has positive effects on bereaved emotion regulation, grief alleviation, and increase of mindfulness score as significant differences were observed for the comparisons of scores between post-MBCT and pre-MBCT. Furthermore, in order to examine the relationship between mindfulness and affective reactivity, correlation analyses were conducted between the FFMQ scale and post-MBCT intervention negative emotion state, which includes TRIG-Present, GAD-7, Depression, DERS scales. The results indicate that the mindfulness state of post was highly negatively correlated with all negative emotion state, TRIG-Present *r* = -0.52, *p* < 0.05; GAD-7 *r* = -0.70, *p* < 0.001; Depression *r* = -0.59, *p* < 0.01; DERS *r* = -0.91, *p* < 0.001.

### Behavioral Results

Accuracy and RT of all participants were recorded while they performed the Stroop task in the scanner. The accuracy did not present significant difference across MBCT by Mann–Whitney test, *ns* (pre-MBCT accuracy = 86.2% and post-MBCT accuracy = 86.8%). We found that RT to congruent trials (in both numerical magnitude and physical size) was not reliably different between pre- and post-MBCT, *t*(37) = 0.59, *ns*, whereas a significant reduction in RT to incongruent trials, *t*(37) = -2.4, *p* < 0.05. Significant interaction effect of RT was found in intervention × condition, *F*(1,18) = 6.73, *p* < 0.018. Furthermore, an additional comparison of the proportional interference scores, unbiased by differences in base RT, showed significant reduction after MBCT intervention (from 13.1 to 8.7%), *p* = 0.002. Details were listed in Table 2.

**Table 2:** Comparison of reaction time and percentage interference between pre- and post-MBCT on grief bereavement.

Source	Congruent		Incongruent		*% Interference*	
	*Mean*	*SE*	*Mean*	*SE*	*Mean*	*SE*
Pre-MBCT	549.8	11.4	623.8	13.9	13.1	1.4
Post-MBCT	564.5	10.6	608.0	11.7	8.7	1.8
Significance	Interaction:				Mann–Whitney^†^	
	*F*(1,18) = 6.73, *p* = 0.018^∗^				*z* = -2.33, *p* = 0.002^∗∗^	

*^†^Mann–Whitney test was used instead of paired t-test because of the normality violation. ^∗^p < 0.05; ^∗∗^p < 0.01.*

### Neuroimage Results on Numerical Stroop Task

**FIGURE 1:**
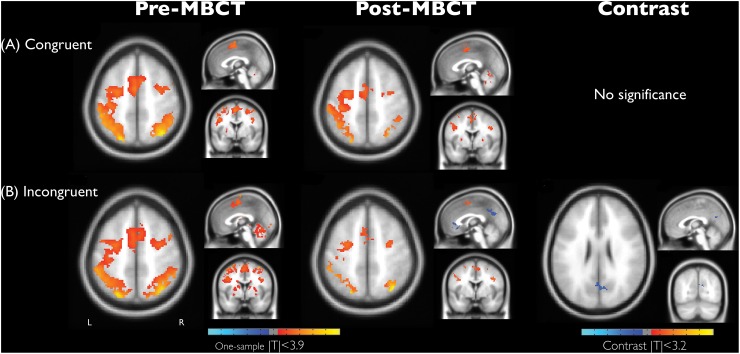
Brain activation maps associated with condition effect of **(A)** congruency and **(B)** incongruency and its difference and the intervention effect (before and after MBCT) in the numerical Stroop task on grief bereavement (AlphaSim corrected *p* < 0.05). Anatomical images were shown with coordinate location = [0, 0, 52] for one-sample t maps and [–2, –70, 26] for contrast maps. L, the left hemisphere.

**FIGURE 2:**
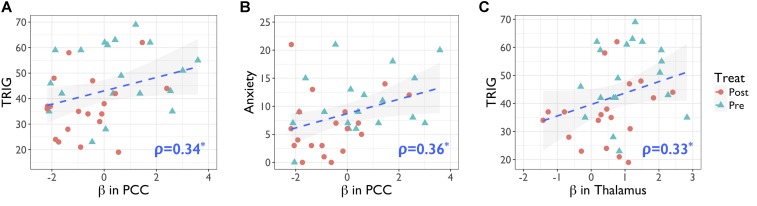
Positive associations of neuropsychological tests and the brain activity (beta value) in the incongruent condition of numerical Stroop task. **(A)** PCC activity in numerical Stroop associated with the TRIG score, *r* = 0.34, *p* < 0.04; **(B)** PCC activity in numerical Stroop associated with the Anxiety score, *r* = 0.36, *p* < 0.03; **(C)** thalamus activity in numerical Stroop associated with the TRIG score, *r* = 0.33, *p* < 0.05. Statistical significance was based on Spearman’s ρ. PCC, posterior cingulate cortex; TRIG, Texas Revised Inventory of Grief; Anxiety, Becker Anxiety Inventory.

Figure 1 demonstrates the significant recruitment of the dorsal attention network (DAN) in a single session of performing the numerical Stroop task, and the detailed information of brain regions were shown in Tables 3–5. The DAN activation level was generally reduced after MBCT while preserving the accuracy, but the results of incongruent trials, involving in the higher cognitive-load of inhibition, disclosed significant deactivation in both anterior cingulate cortex (ACC) and posterior cingulate cortex (PCC). Similar to the RT results, the congruent trials did not present MBCT effects in brain recruitments, whereas the brain recruitment to incongruent trials after MBCT only showed significant reduction in right PCC/precuneus under AlphaSim *p* < 0.05. The interaction effect of fMRI results (intervention × condition) showed single negative activation located at PCC, similar as the incongruency outcome. We further evaluated the associations between the neuropsychological assessment and the brain regional activity (PCC and hypothetical thalamus) involved in the incongruent condition of the numerical Stroop task. Figure 2 shows significant correlations (a) between TRIG and PCC; (b) between Anxiety and PCC; and (c) TRIG and thalamus, Spearman’s ρ > 0.33, *p* < 0.05. Figure 2 demonstrates that less grief and anxiety were associated with reduced brain activations of PCC or thalamus involved in the numerical Stroop task.

**Table 3:** Peak locations of brain activities associated with congruent condition during the numerical Stroop task, including before and after the MBCT intervention (AlphaSim corrected *p* < 0.05).

Brain regions		L/R	AAL	*x*	*y*	*z*	*t*-value
***Pre-MBCT (congruent condition)***							
Frontal	Supplementary motor area	L	19	-4	-4	58	8.421
	Middle frontal gyrus	R	8	46	0	56	6.288
	Inferior frontal gyrus	R	12	56	14	20	7.161
		R	14	38	28	30	5.767
Parietal	Inferior parietal lobule	L	61	-28	-56	44	9.687
	Angular gyrus	R	66	30	-54	46	8.515
Occipital	Inferior occipital cortex	L	53	-18	-90	-8	8.131
Cerebellum		R	98	16	-50	-20	9.417
		L	99	-24	-64	-28	7.738
		R	104	18	-60	-46	7.11
		L	91	-8	-76	-24	6.67
Subcortical	Thalamus	L	77	-16	-12	10	6.117
***Post-MBCT (congruent condition)***							
Frontal	Precentral gyrus	L	1	-30	-28	56	9.066
		R	2	56	12	36	6.216
		R	2	22	-6	54	5.211
	Supplementary motor area	L	19	-4	-4	56	6.113
Parietal	Angular gyrus	R	66	28	-58	42	6.154
	Inferior parietal lobule	R	62	38	-44	56	5.584
Occipital	Lingual gyrus	R	48	24	-88	-6	8.097
	Middle occipital gyrus	L	51	-40	-86	-4	7.599
Temporal	Superior temporal gyrus	L	81	-50	-40	24	6.676
	Inferior temporal gyrus	R	90	52	-44	-10	5.633
Cerebellum		R	98	20	-48	-22	8.313
		L	99	-20	-60	-30	6.959
Subcortical	Thalamus	L	77	-14	-22	6	6.27
	Caudate nucleus	R	72	18	-8	26	4.793
	Putamen	R	74	26	2	16	4.781

**Table 4:** Peak locations of brain activities associated with incongruent condition during the numerical Stroop task, including before and after the MBCT intervention (AlphaSim corrected *p* < 0.05).

Brain regions		L/R	AAL	*x*	*y*	*z*	*t*-value
***Pre-MBCT (incongruent condition)***							
Parietal	Inferior parietal lobule	L	61	-40	-50	50	9.243
	Postcentral gyrus	R	58	58	-20	32	8.198
Occipital	Inferior occipital cortex	R	54	28	-88	-2	10.584
Cerebellum		R	104	32	-52	-56	8.916
		R	104	20	-66	-46	6.613
***Post-MBCT (incongruent condition)***							
Frontal	Supplementary motor area	R	20	6	0	56	6.228
	Anterior cingulate gyrus	L	31	-2	38	0	-7.867
	Precentral gyrus	L	1	-34	-26	56	7.031
		R	2	44	6	28	6.268
		L	1	-32	-4	56	5.785
		R	2	28	-8	52	5.619
		L	1	-48	0	38	5.382
	Insula	R	30	32	16	12	6.831
		L	29	-36	10	10	6.269
Parietal	Superior parietal lobule	L	59	-24	-62	42	6.808
	Rolandic operculum	R	18	40	-20	22	-6.73
	Angular gyrus	R	66	30	-54	44	6.209
	Precuneus	L	67	-4	-62	28	-9.153
		R	68	6	-52	30	-7.538
Occipital	Inferior occipital cortex	L	53	-22	-94	-8	6.681
Cerebellum		R	100	28	-64	-26	6.227
Subcortical	Caudate nucleus	L	71	-18	-18	22	6.984

**Table 5:** Peak locations of brain regions showing intervention differences in brain activity during incongruent condition of numerical Stroop task (AlphaSim corrected *p* < 0.05).

Brain regions		L/R	AAL	*x*	*y*	*z*	*t*-value
***Pre-MBCT > Post-MBCT (incongruent condition)***							
Parietal	Precuneus/Cuneus	R	68/46	10	-62	22	4.256

## Discussion

The purpose of the current study was to investigate the facilitative effect of an 8-week MBCT on emotion regulation and executive function in bereavement grief. As being taught to acknowledge all emotion, thoughts, and body sensations with non-judgmental acceptance, the bereaved participants attempted to avoid being engulfed by the catastrophic stories of what these cognition associated with them (Kabat-Zinn, 1994). As predicted, the bereaved participants after MBCT intervention reported a significant decrease in grief, anxiety, depression, and difficulties in emotion regulation, as well as increases in the mindfulness state. These positive changes may arise from the fact that bereaved participants foster a monitoring ability on their grief reactions with emotional acceptance, non-judgmental attitude and switching attention back to the present moment. Such practices over time may sharpen the participants’ skills of emotion regulation in their daily life, allowing them to relax physically. From the group discussion, many bereaved reported they have had better sleep and felt more vitality in their daily life after the MBCT intervention.

Our first aim is to study the cognitive improvements following the grief mitigation following the MBCT intervention. The Stroop task is a cognitive measure to probe the executive control function, which allows people to overcome impulses and override automatic behavior. In this work, we replaced the traditional color-word Stroop task with the numerical Stroop task for the bereaved participants considering their inability to handle high cognitive load in the beginning phase (Huang et al., 2012). Following the mindfulness training, we noticed the reduced interference RT score when performing the numerical Stroop task in the bereaved participants, indicating the improved cognitive control performances (Colcombe et al., 2005) associated with the declined grief level following the MBCT. Previously, Teper and Inzlicht (2013) and Teper et al. (2013) demonstrated higher emotional acceptance and better performance in the EEG-based Stroop task among mindfulness meditators, suggesting mindfulness trainings such as meditative practices facilitate the executive control. Gallant (2016) also supported this statement in their review article (Gallant, 2016). Furthermore, even though Teper and Inzlicht (2013) conjectured that mindfulness practices promoted executive control preceding the emotion-regulation improvements. However, we could not verify the conjecture based on our observations in bereaved population. Further experimental design is warranted to prove this notion.

Secondly, practicing mindfulness allowed the bereaved individuals to stop overwhelming spontaneous ruminations and decrease the emotional interferences over the cognitive functions. Therefore, we used the fMRI experiment to disclose the underlying neurophysiological basis of MBCT-based cognitive improvements in bereavement grief. Results showed the numerical Stroop activation of bilateral DAN, encompassing the middle frontal gyrus and superior parietal gyrus, similar to the previous report (Huang et al., 2012). Overall, after the MBCT intervention, the reduced DAN activity implied the less cognitive loads involved in the numerical Stroop task. Meanwhile, the deactivations of ACC and PCC in the bereaved participants became prominent when dealing with the incongruent trials, indicating a cross-network interaction in the task. Interestingly, the default-mode network (DMN) deactivation was previously noticed in coping with high cognitive loads of working memory tasks (Liang et al., 2016). We conjectured that the mismatches originate from a hyper-activity of DMN with excessive internal thoughts in the bereaved population, thereby intervening their normal functions in cognitive performances. Similarly, Gündel et al. (2003) reported the activities of PCC and medial frontal gyrus among the bereaved women responding to grief-related words, indicating these regions are involved in the affective processing. Christoff et al. (2016) in her review elaborated the role of DMN core in internal and spontaneous thoughts, impacting the conceptual and emotional processing. In our report, the PCC activity in the numerical Stroop task had significant positive correlation with TRIG and Anxiety, revealing that strong grief level associates with the positive beta value of PCC performing the numerical Stroop task. At last, we did not find the hypothetical correlation between T-FFMQ and PCC/thalamus activity. One possibility is that DMN-related regions can be more involved in the spontaneous activities of emotional arousal, rather than participating in the mindfulness directly. The other possibility is that the mindfulness dosage of the conventional 8-week MBCT protocol may be insufficient for relieving emotion disentanglements in bereavement grief. Further MBCT-based studies are warranted to verify the speculation on the bereaved population.

Though the current results suggest that MBCT practices leads to enhanced emotion regulation in subjective assessments and executive control in fMRI environment, we did not probe the brain activity involved in the emotion arousal. To the humanity concerns, we attempt to avoid raising extra affective arousal to the bereaved participants. Since our study did not employ a direct measure of emotional sensitivity, it is difficult to say whether DMN and thalamus were directly involved in affective processing; however, the literature gave strong support on the emotional involvement of both PCC, ACC and thalamus in major depressive disorder, insomnia and anxiety disorders (Greicius et al., 2007; Bastien, 2011; Christoff et al., 2016). Secondly, due to the individual differences of participation timing, initial sadness degree and comprehension ability on mindfulness, the learning curve to achieve the expected mindfulness was diverse. For example, some of them had reported being able to relate their situations in a mindful way in the final session of the MBCT course, but some reported that they felt better only when they were inside of the group. Therefore, the emotional stability in the same MBCT group exhibited strong inter-subject variability, imposing one of the confounding factors on the final assessments. Third, Huang et al. (2012) used the numerical magnitude and physical size of the numerical Stroop task to differentiate the hemispheric laterality between the elderly and youth; however, we did not notice the laterality changes in the current work.

Additionally, one practical challenge in this study was the recruitment of a control group without treatments, due to the fact that none of the recruited bereaved participants was willing to be enrolled in the control group. Nevertheless, the enrollment of the active control might be bypassed, because literature disclosed that the grief perception measured by TRIG after the bereavement point took multiple years to recover back to a normal status, far from 8 weeks of MBCT (Zisook et al., 1982). Similarly, Tseng et al. (2017) reported Taiwanese bereavements took approximately 4 years to restore the depression scale to the level prior to the bereavement. The evidence demonstrated that the grief bereavements have long-term impacts without treatments and are not easily alleviated within 8 weeks. Further investigations are warranted to support the statements on the topic of grief bereavements.

Conclusively, practicing the 8-week mindfulness training allowed significant alleviation of grief, anxiety, depression, and elevated their mindfulness state in bereaved individuals. The mindfulness training not only benefited emotion regulation, but also reduced the emotional interferences over cognitive functions. Results revealed the reduced interference RT score in the numerical Stroop task among the bereaved participants, indicating improved executive control function. Furthermore, the positive brain activities of PCC and thalamus showed the intervening effect on the Stroop task, but the intervening effects of PCC and thalamus were reduced following the MBCT intervention, associated with the reduced bereavement grief. Based on the beneficial effects of MBCT intervention, we encourage the bereaved population to practice mindfulness training to avoid overwhelming emotional arousal and to preserve the quality of daily life.

## Author Contributions

F-YH, CW, Y-PC, and C-MH contributed to the conception and experiment design. F-YH, A-LH, and CW contributed to preparation and revision of the manuscript. CW and L-MH contributed to data analysis. J-ST and T-JH contributed to clinical evaluation of mental health. All authors reviewed and approved the manuscript.

## Conflict of Interest Statement

The authors declare that the research was conducted in the absence of any commercial or financial relationships that could be construed as a potential conflict of interest.
